# Ethical, social, and cultural issues related to clinical genetic testing and counseling in low- and middle-income countries: protocol for a systematic review

**DOI:** 10.1186/s13643-017-0535-2

**Published:** 2017-07-11

**Authors:** Adrina Zhong, Benedict Darren, Helen Dimaras

**Affiliations:** 1 Division of Health Promotion, University of Toronto, Toronto, Ontario Canada; 2 Division of Clinical Public Health, Dalla Lana School of Public Health, University of Toronto, Toronto, ON Canada; 3 Human Biology Program, Faculty of Arts & Science, University of Toronto, Toronto, ON Canada; 4 Department of Ophthalmology and Vision Sciences, Faculty of Medicine, University of Toronto, Toronto, Ontario Canada; 5 Department of Ophthalmology and Vision Sciences, The Hospital for Sick Children, 555 University Ave, Room 7260, Toronto, Ontario M5G 1X8 Canada; 6 Child Health Evaluative Sciences, SickKids Research Institute, Toronto, Ontario Canada; 7 Department of Human Pathology, College of Health Sciences, University of Nairobi, Nairobi, Kenya; 8 Present Address: Michael G. DeGroote Medical School, McMaster University, Hamilton, Ontario Canada

**Keywords:** Genetic testing, Global health, Health equity, Genetic counseling, Ethics, Society, Culture

## Abstract

**Background:**

There has been little focus in the literature on how to build genetic testing and counseling services in low- and middle-income countries in a responsible, ethical, and culturally appropriate manner. It is unclear to what extent this area is being explored and what form further research should take. The proposed knowledge synthesis aims to fill this gap in knowledge and mine the existing data to determine the breadth of work in this area and identify ethical, social, and cultural issues that have emerged.

**Methods/design:**

An integrated knowledge translation approach will be undertaken by engaging knowledge users throughout the review to ensure relevance to their practice. Electronic databases encompassing various disciplines, such as healthcare, social sciences, and public health, will be searched. Studies that address clinical genetic testing and/or counseling and ethical, social, and/or cultural issues of these genetic services, and are performed in low- and middle-income countries as defined by World Bank will be considered for inclusion. Two independent reviewers will be involved in a two-stage literature screening process, data extraction, and quality appraisal. Studies included in the review will be analyzed by thematic analysis. A narrative synthesis guided by the social ecological model will be used to summarize findings.

**Discussion:**

This systematic review will provide a foundation of evidence regarding ethical, social, and cultural issues related to clinical genetic testing and counseling in low- and middle-income countries. Using the social ecological model as a conceptual framework will facilitate the understanding of broader influences of the sociocultural context on an individual’s experience with clinical genetic testing and counseling, thereby informing interdisciplinary sectors in future recommendations for practice and policy.

**Systematic review registration:**

PROSPERO CRD42016042894

**Electronic supplementary material:**

The online version of this article (doi:10.1186/s13643-017-0535-2) contains supplementary material, which is available to authorized users.

## Background

Clinical genetic testing is a medical test that analyzes human DNA to detect anomalies that may have pathological consequences. It has become part of the standard care to diagnose and manage several hundred inherited disorders [1]. Coupled with genetic counseling services, which aim to assist patients and families to cope with and understand the results of testing, the aim is to predict the chance of developing disease or transmitting disease-causing variants to offspring. As technology has evolved and become increasingly more cost-effective, genetic testing services have been introduced in low- and middle-income countries (LMICs), usually through research initiatives [2–7] or formal international partnerships [8–10]. However, the current ability to perform genetic testing in LMICs appears to have surpassed the availability of genetic counseling, which is largely a Western concept and profession. Indeed, the number of genetic counselors available globally is far lower than the need for their services, meaning that physicians bear much of the counseling responsibility [11–13]. In LMICs, where physicians have higher patient loads and often very limited training in medical genetics, it is challenging to effectively support patients and families with interpretation and coping with the diagnosis.

While there has been a push to bring genomic science from “lab to village” [14], there is little focus in the literature on how to build genetic testing and counseling services in LMICs in a responsible, ethical, and culturally appropriate manner. Much of the global health ethics discussion for genetics has centered on informed consent for genomic research on vulnerable populations [15] or the ethical use of data from genomic studies on ethnic diversity (culture, identity, and genes) [16–20]. Studies reporting on development of genetic diagnostic services in LMICs have largely commented on capacity building, technical success, and sustainability of the health service [4, 5, 10, 21–26]. The World Health Organization’s Human Genetics (HGN) endorses ethical conduct concerning research, access, and affordability of genetic services and guides regulations governing genetic patents, databanks, and pharmacogenomics [27]; however, little guidance exists on the ethics and social issues of implementation of genetic testing and counseling in LMICs.

Acknowledging the diversity of LMICs and their sociocultural contexts, introducing clinical genetic services can create uniquely complex issues that differ depending on local setting. Experts in the field are beginning to recognize the urgent need for a thoughtful and ethically sound approach to implementing genetic services in LMICs so that the unique needs of those patient populations are met [28, 29], and several independent studies are beginning to bring ethical and sociocultural issues in genetics to the forefront [30–33]. However, it is unclear to what extent this area is being explored and what form further research should take. The proposed knowledge synthesis aims to fill this gap in knowledge and mine the existing data to determine the breadth of work done in this area and identify ethical, social, and cultural issues that have emerged to ultimately define areas of new investigation in this area.

To our knowledge, a systematic review on our proposed topic has not previously been performed. A preliminary search using MEDLINE reveals several studies on implementation of disease-specific genetic services and/or counseling for various genetic disorders (e.g., beta thalassemia [34], sickle cell anemia [10, 23, 30, 35–38], retinoblastoma [3, 4, 6], Huntington’s disease [22], muscular dystrophy [39], deafness [40]) as well as reports on national genetic screening programs [17, 21]. The National Institutes of Health supports researchers studying the ethical, legal, and societal implications of genomic research in sub-Saharan Africa [41], but there is no equivalent push for similar research regarding the implementation of clinical genetic testing services, despite the fact that existing medical genetic services established in North America and Europe are being exported to LMICs. Although some genetic services have had long-standing and successful implementation in LMICs, such as national screening programs for sickle cell anemia, the range of clinical genetic services offer a variety of challenges to implementation. The diversity in genetic services and local sociocultural contexts lead to complex ethical, social, and cultural issues, which may not apply to all genetic services nor all LMICs.

Our proposed systematic review is topical and very timely, given the increasing trend to bring forefront science to healthcare in the so-called developing world, and is essential to define the extent of knowledge in the field and identify knowledge gaps and new research directions to the needs of this unique patient population which are beginning to be adequately addressed.

## Methods/design

### Protocol and registration

This protocol adheres to the Preferred Reporting Items for Systematic Review and Meta-Analysis Protocols (PRISMA-P) 2015 statement [42] (Additional file 1). The final review will be reported according to the PRISMA guidelines [43]. This protocol is registered with the PROSPERO International Prospective Register of Systematic Reviews [44] (registration number CRD42016042894).

### Objective

This knowledge synthesis will use a systematic review with narrative synthesis to answer the following research question: what are the ethical, social, and cultural issues related to clinical genetic testing and counseling in LMICs? Specifically, this study will aim to (1) determine the extent, range, nature, and outcomes of research; (2) identify gaps in the existing literature; and (3) identify key research priorities surrounding the ethical, social, and cultural issues related to the implementation of clinical and medical genetic services in LMICs.

### Engagement of knowledge users

An “integrated knowledge translation” approach will be used by engaging with stakeholders (end users) throughout the research process [45]. An end-user committee will be formed by representatives from a variety of knowledge user groups, including clinicians, former patients with lived experience, and family support advocates from around the world. Partnering with this committee will ensure our systematic review responds to the information needs of knowledge users in that the topic is relevant and our findings are tailored to facilitate knowledge translation for future policy and practice. End users hold positions on organizations dedicated to improving care for genetic disorders in LMICs (e.g., Kenya National Retinoblastoma Strategy group; World Eye Cancer Hope), with experience transforming evidence into action at institutional and national levels. The end-user committee will meet three times over the course of the systematic review to (1) discuss proposed protocol, search strategies, and selection criteria; (2) review data collected and discuss data analysis approach; and (3) review synthesized results and extrapolate recommendations for future policy and practice.

### Search strategy

The search strategy will be developed with the assistance of an information scientist. The following major databases will be searched: MEDLINE, Embase, Web of Science, PsycINFO, CINAHL, and LILACS. Additionally, the following specialist registers will be searched: CCRCT, CDSR, DARE, BiblioMap, and HealthPromis. These databases have been selected to include a variety of disciplines, such as healthcare, social sciences, psychology, and public health. A sensitive database search strategy will be developed for MEDLINE using controlled vocabulary (MeSH) and free-text terms to combine the concepts of “genetic testing and/or counseling” and “LMICs.” The concept of “ethical, social, and cultural factors” will be omitted from our search strategy to ensure that there are no missed papers, as this third concept is quite vast. Methodological filters for study type will not be included since these tend to decrease the sensitivity of database searches. A sample MEDLINE search strategy is included in Additional file 2.

To supplement database searches, additional peer-reviewed published studies will be identified through hand-searching topical journals, bibliographies of relevant primary and review articles, and relevant studies recommended by the end-user committee, as well as additional experts in genetic services. All citations retrieved by these supplementary methods will be compiled into EPPI-Reviewer and screened for appropriateness against the inclusion and exclusion criteria.

### Selection criteria

This review will address the scope of the literature on the inclusion of the following three conceptual components: (1) clinical genetic testing or genetic counseling services (where genetic counseling refers to both formal genetic counseling as well as other forms of communication from healthcare professional to patient regarding heritability and/or genetics of a disorder); (2) studies on populations in LMICs as defined by the World Bank [46]; and (3) ethical, social, and/or cultural factors which influence the implementation of genetic testing and/or counseling (e.g., socioeconomic status, level of education, religion, culture, attitudes, ethical considerations). All primary research studies presenting the above criteria in quantitative or qualitative data and secondary research studies designed as a discussion, personal narrative, or review paper will be included.

Studies performed in high-income countries (and/or states/territories, as in the case of Hong Kong and Taiwan), as defined by the World Bank [47, 48], will be excluded. Studies focused solely on the technological aspect of genetic testing (e.g., development and/or application of a novel technique), and studies related to basic genomic research (e.g., those looking at migration, ethnicity, or genomics of populations), will also be excluded. We will exclude studies in languages other than English for practical reasons. Studies published before 1990 will be excluded to limit findings to more recent publications.

### Study selection

#### Initial screening

Results of the searches from different search strategies will be managed on EPPI-Reviewer 4 software for managing and analyzing data in research synthesis (Evidence for Policy and Practice Information and Coordinating Centre, UK). This software will import references from database searches and discard any duplicates.

In EPPI-Reviewer, the inclusion/exclusion criteria will be used to create a set of codes (i.e., coding tool) that can be applied to each study. This coding tool will be initially applied to titles and abstracts of retrieved studies, and studies that meet the inclusion criteria will proceed to the second screening stage. Each article will be reviewed by two independent reviewers, and a third will settle discrepancies.

#### Second screening

For studies that pass initial screening, full-text articles will be retrieved and uploaded in EPPI-Reviewer. Study authors will be contacted if articles are not easily accessible; if we are unable to retrieve an article, then it will be excluded. Using the coding tool, the inclusion/exclusion criteria will be applied to full-text articles by two independent reviewers and a third will settle discrepancies.

### Data extraction

A data extraction form will be developed using Microsoft Excel spreadsheet (Microsoft Corp., Redmond, WA, USA) to pull information of interest from the included full-text articles. The form will be pilot-tested on a randomly selected subset of included articles and modified as necessary before proceeding with the remaining articles. Data extraction of all included articles will be performed by the lead reviewer. To reduce bias and mistakes during data extraction, a second reviewer will independently extract 10% of articles to check for consistency and accuracy of the data collected [49]. A third reviewer will settle any discrepancies.

The data extraction form will collect information on bibliographic details (country, setting, year of publication, funding sources); study design and methods; participant characteristics (inclusion and exclusion criteria, age range, gender); intervention details; results; and outcomes of interest (ethical, social, and cultural factors related to genetic testing or counseling).

### Quality assessment

Quality appraisal will be performed using the “QUALSYST” quality assessment tool developed by the Alberta Heritage Foundation for Medical Research [50]. This tool contains 14 items for quantitative studies and 10 items for qualitative studies that are scored depending on the degree to which each criteria was met (2 points for “yes,” 1 point for “partial,” and 0 points for “no”). Criteria items not applicable for a given study are noted and excluded from calculation of the summary score. Quality appraisal will be undertaken by the lead reviewer, and a second reviewer will independently appraise 10% of the included studies to confirm consistency of appraisal ratings [49]. A third reviewer will settle any discrepancies. Quality scores will not be used to exclude studies but to identify the overall quality of the included studies and their strengths/weaknesses.

### Data synthesis

It is anticipated that the study criteria and selection procedure will capture a great diversity of research methods in the included articles. Therefore, a narrative synthesis is an appropriate way to report the findings, as this approach is suitable for both quantitative and qualitative studies, and the purpose is to summarize and explain the findings using primarily text [51]. Using a narrative synthesis will capture a multidisciplinary and methodologically diverse group of research in the topic area of ethical, social, and cultural issues related to genetic services in LMICs.

The Economic and Social Research Council has established guidelines and a general framework to reporting a narrative synthesis that consists of four elements [51]: (1) development of a theory of why and how an intervention works, (2) preliminary synthesis of findings, (3) exploration of relationships within and between studies, and (4) assessment of the robustness of the synthesis.

#### Element 1: development of a theory

Since this systematic review will not exclusively synthesize intervention studies, the development of a theory is not applicable.

#### Element 2: preliminary synthesis of findings

The extracted data will be input verbatim into NVivo-11 (QSR International), a software which facilitates qualitative analysis of textual data. We will employ an inductive (“bottom up”), realist analysis to generate descriptive codes and apply them to meaningful data points. This preliminary description of findings will be subject to a further thematic analysis, identifying common patterns and issues among all included studies [52].

#### Element 3: exploring relationships within and between studies

**Fig. 1:**
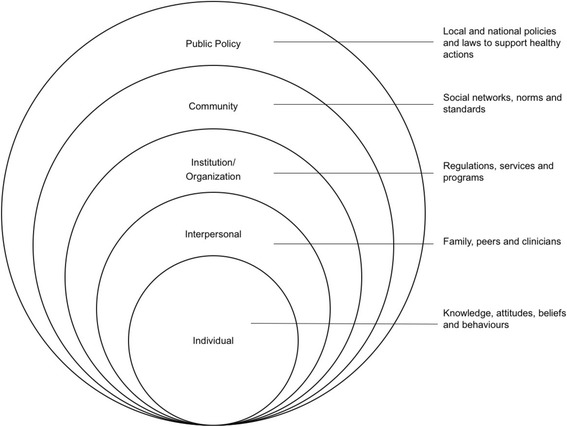
Social ecological model. The social ecological model shows the levels of influence on individual health outcomes, which ranges from the individual to interpersonal relationships, institutions, community, and public policy. Adapted from the Centers for Disease Control and Prevention [54]

To explore relationships across all studies, themes that emerge from the preliminary synthesis will be mapped onto the social ecological model, a conceptual framework that is often used to describe possible etiology of health issues and set the groundwork for future interventions [53]. The social ecological model is based on ecological systems theory and proposes that individual health outcomes are influenced by interactions with larger environmental, social, and cultural context [53]. In this narrative synthesis, the social ecological model will frame identified ethical, social, and cultural issues related to genetic services in LMICs on concentric levels of influence including individual, interpersonal, organizational/institutional, community, and public policy [53, 54] (Fig. 1). This model will facilitate the analysis of intra-level and inter-level relationships of these issues. Additionally, this model will highlight where the evidence lies currently and identify gaps for future research.

#### Element 4: assessment of robustness of synthesis

A quality appraisal will be performed on all included studies, and an overall assessment of the strength of the evidence will be determined. An accompanying description addressing the robustness of the evidence in the narrative synthesis will be provided. The strength of the evidence is pertinent to drawing future recommendations for policy/practice of genetic services in LMICs.

## Discussion

### Strengths and limitations

A knowledge synthesis of ethical, social, and cultural issues pertaining to genetic testing and counseling in LMICs is needed to build understanding of the factors that are relevant in implementing genetic services. This synthesized evidence is pertinent to stakeholders in developing appropriate genetic services in LMICs. In addition, the evidence of these ethical, social, and cultural factors are not exclusively relevant to LMICs; human experiences of genetic services may be universal and issues identified in this review may also pertain to high-income countries, where clinical genetic services have become the standard care.

A strength in this narrative synthesis is the systematic and transparent approach throughout the research process by using validated methods and guidelines. Engagement of knowledge users at multiple stages of the study ensures that their input and lived experiences guide the direction of research, such that the end product will be useful to their practice. The involvement of two reviewers during study screening, data extraction, and quality appraisal will ensure reliability of conclusions. Moreover, the heterogeneous nature of the methodological approaches in this narrative synthesis captures a large variety of ethical, social, and cultural factors in order to paint a holistic image of the genetic testing or counseling experience in LMICs.

A narrative synthesis guided by the Social Ecological Model facilitates acknowledgement and understanding of ethical, social, and cultural factors in genetics in a systemic and interactive manner. All levels of influence of the Social Ecological Model must be considered when developing appropriate genetic services, and this narrative synthesis provides the evidence based to further research and development of policy and practice recommendations.

A limitation of this review is the exclusion of non-English literature, due to practical reasons. By limiting the narrative synthesis to English studies only, it excludes the additional useful insights that are present in local languages in LMICs. Similarly, there could be useful insights in gray literature in LMICs. Ultimately, this review is an important first step to build evidence for knowledge users within multiple sectors and stimulate interdisciplinary discussion with the overall aim of developing evidence-based, ethical, and culturally sensitive clinical genetic services in LMICs.

### Dissemination

The review team plans to engage the broader medical genetics community to disseminate study results and initiate knowledge exchange. Dissemination among the scientific community will be achieved through publication in a peer-reviewed journal and presentation at international scientific conferences about genetics and global health. The synthesized findings of this systematic review will be shared with and evaluated and implemented by the end-user committee. They will disseminate the results to their client base, practice, academic partners, and/or organization audience and facilitate translation to relevant decision-makers.

## Additional files

Additional file 1: PRISMA-P checklist. Items addressed in a systematic review protocol. (DOC 83 kb)

Additional file 2: Search strategy. Sample MEDLINE search strategy. Sample search strategy for MEDLINE. (DOCX 135 kb)

## Abbreviation

LMICs: Low- and middle-income countries
