# Supplementary material for: Ethical, social, and cultural issues related to clinical genetic testing and counseling in low- and middle-income countries: protocol for a systematic review
Source: Syst Rev. 2017 Jul 11;6:140. doi: 10.1186/s13643-017-0535-2 (PMC5505010; doi:10.1186/s13643-017-0535-2)
Supplement: Supplementary file 2 — Search strategy. Sample MEDLINE search strategy. Sample search strategy for MEDLINE. (DOCX 135 kb) [file 13643_2017_535_MOESM2_ESM.docx]

# Additional file 1: Sample MEDLINE search strategy

Database: Ovid MEDLINE(R) 1946 to Present with Daily Update, Ovid MEDLINE(R) In-Process & Other Non-Indexed Citations <July 12, 2013>

Search Strategy:

1 (Afghanistan or Albania or Algeria or Angola or Antigua or Argentina or Armenia or Azerbaijan or Bangladesh or Barbuda or Belarus or Belize or Benin or Bhutan or Bolivia or Borneo or Bosnia or Botswana or Brazil or Bulgaria or "Burkina Faso" or Burundi or Cambodia or Cameroon or "Cape Verde" or "Central African Republic" or Chad or Chile or China or Colombia or Comoros or Congo or "Costa Rica" or "Cote d'Ivoire" or Cuba or Djibouti or Dominica or "Dominican Republic" or Ecuador or Egypt or "El Salvador" or Eritrea or Ethiopia or Fiji or "French Guiana" or Gabon or Gambia or Gaza or Georgia or Ghana or Grenada or Grenadines or Guadeloupe or Guatemala or Guinea or Guyana or Haiti or Hercegovina or Herzegovina or Honduras or India or Indonesia or Iran or Iraq or "Ivory coast" or Jamaica or Jordan or Kazakhstan or Kenya or Kiribati or Korea or Kosovo or "Kyrgyz Republic" or Kyrgyzstan or Laos or Latvia or Lebanon or Lesotho or Liberia or Libya or Lithuania or Macedonia or Madagascar or Malawi or Malaysia or Maldives or Mali or "Marshall Island*" or Martinique or Mauritania or Mauritius or "Mekong Valley" or Mexico or Micronesia or Moldova or Mongolia or Montenegro or Morocco or Mozambique or Myanmar or Namibia or Nepal or "Netherlands Antilles" or Nevis or Nicaragua or Niger or Nigeria or Pakistan or Palau or Panama or "Papua New Guinea" or Paraguay or Peru or Philippines or Romania or "Russian Federation" or Rwanda or "Saint Kitts" or "Saint Lucia" or "Saint Vincent" or Samoa or "Sao Tome and Principe" or Senegal or Serbia or Seychelles or "Sierra Leone" or "Solomon Island*" or Somalia or "South Africa" or "Sri Lanka" or "St. Lucia" or "St. Vincent" or Sudan or Suriname or Swaziland or Syria or Taiwan or Tajikistan or Tanzania or Thailand or Tibet or Timor or Togo or Tonga or Tunisia or Turkey or Turkmenistan or Tuvalu or Uganda or Ukraine or Uruguay or Uzbekistan or Vanuatu or Venezuela or Vietnam or "West Bank" or Yemen or Zambia or Zimbabwe).mp. (1001728)

2 ("developing area" or "developing countr*" or "developing nation*" or "developing world" or "least developed countr*" or "least developed nation*" or "less developed nation*" or "less developed countr*" or LMIC or "resource-poor countr*" or sahel or "third world countr*" or "third world nation*" or "under developed countr*" or "under developed nation*" or "underdeveloped countr*" or "underdeveloped nation*").mp. (102667)

3 Developing Countries/ (62407)

4 or/1-3 (1047239)

5 genetics, medical/ or genetic counseling/ (24424)

6 Genetic Testing/ (26387)

7 exp Prenatal Diagnosis/ (58934)

8 Genetic Privacy/ (1589)

9 ("antenatal diagnos*" or "antenatal screening*" or "chorionic villi biops*" or "chorionic villi sampl*" or "chorionic villus sampl*" or "clinical genetic*" or "fetal ultrasonograph*" or "fetal ultrasound*" or "genetic counsel*" or "genetic privacy" or "genetic test*" or "human genetic*" or "intrauterine diagnos*" or "maternal serum screening*" or "maternal serum test*" or "medical genetic*" or "nuchal fold" or "nuchal translucency" or "placental function test*" or "preimplantation diagnos*" or "prenatal diagnos*" or "prenatal screening" or "prenatal ultrasonic diagnos*" or "prenatal ultrasonograph*" or "prenatal ultrasound*" or "preventive genetic*" or amniocentes* or amnioscop* or embryoscop* or eugenesis or eugenic* or fetoscop* or "amnion centesis").mp. (108532)

10 (amniotic fluid adj2 (aspirat* or analys*)).mp. (610)

11 or/5-10 (135048)

12 4 and 11 (6863)
